# Two year effects of food allergen immunotherapy on quality of life in caregivers of children with food allergies

**DOI:** 10.1186/1710-1492-10-57

**Published:** 2014-11-25

**Authors:** Stefania Arasi, Iris M Otani, Erik Klingbeil, Philippe Bégin, Clare Kearney, Tina LR Dominguez, Whitney M Block, Geraldine O’Riordan, Kari C Nadeau

**Affiliations:** Stanford Alliance for Food Allergy Research, Palo Alto, CA USA; Department of Pediatrics, Division of Immunology and Allergy, Stanford University, 269 Campus Drive, CCSR Building Suite 3215, Stanford, CA 94305 USA

**Keywords:** Food allergen immunotherapy, Food allergy, Quality of life, Health-related quality of life

## Abstract

**Background:**

Food allergy (FA) can have serious psychosocial and economic repercussions on food-allergic children and their caregivers and be associated with negative effects on their quality of life. Food allergen immunotherapy (IT) is a promising experimental therapy but can be linked to anxiety. This study investigated the effects of IT on FA-specific health-related quality of life (HRQL) over a 24 month-follow-up in caregivers of children with single and multiple food allergies. We hypothesized that characteristics such as age, asthma at baseline and respiratory allergic reactions during therapy were key characteristics that influenced HRQL scores.

**Methods:**

A validated Food Allergy Quality of Life – Parental Burden Questionnaire (FAQL-PB) was used to assess HRQL. It was randomly distributed to and filled out by caregivers of 57 food-allergic children enrolled in clinical trials of IT. The same parent answered the FABQL-PB questionnaire at baseline and for 6-month, 12- month, 18- month, and 24-month time points on IT.

**Results:**

Caregiver HRQL improved significantly (change < - 0.5, p <0.0001) at each follow-up time point compared to baseline. The percentages of caregivers with improvement in HRQL progressively increased (92% at 24 month-follow-up time point compared to baseline). HRQL improved more in caregivers of participants older than 10 years or desensitized to more than 4 food allergens than those who were not (p <0.0001). Caregivers of participants with pre-existing asthma or dose-related respiratory allergic reactions had less improvement in HRQL than those who did not (p <0.01).

**Conclusion:**

IT lead to improvement in caregiver HRQL. Certain characteristics were associated with greater improvements in caregiver HRQL.

**Electronic supplementary material:**

The online version of this article (doi:10.1186/1710-1492-10-57) contains supplementary material, which is available to authorized users.

## Background

Food allergy (FA) is an adverse immunologic response to a dietary antigen. It is estimated that about 6% and 8% of the pediatric population in Europe [1] and in U.S. [2], respectively, are affected. About 1% [1] and 2.4% [2] of European and American children, respectively, have clinical reactivity to more than one food allergen. Currently, the only FDA- and EMA- approved treatment for FA is food allergen avoidance and rescue therapy, including injectable epinephrine [3].

Food allergy may be associated with a negative impact on the quality of life of patients and their families, even more so than other chronic childhood diseases [4–6]. The burden of FA on caregivers (buying special foods, limiting social encounters, time lost from work, changing careers and emergency room visits) has been reported to play a predominant role in the total annual economic burden of FA [7].

Food allergen immunotherapy (IT) is a promising experimental therapy for food allergy [8] but it can be linked to anxiety. The reported reactions during IT are mostly mild but they can also be severe [9, 10]. We hypothesized that baseline characteristics like age, asthma, and respiratory allergic reactions during therapy were key characteristics that influenced HRQL (health-related quality of life) score.

Previously, two studies showed a HRQL improvement with single-allergen IT after desensitization in pediatric patients with milk (n =30) at about 12 months or peanut allergy (n =100) at 6-9 months after therapy began [11, 12]. Our group published a study examining the effects of mIT (IT with multiple food allergens) and rush mIT (mIT with omalizumab treatment) on caregiver HRQL and found significant improved HRQL scores in both groups [13]. However, in the previously published studies there remained several unanswered questions like whether there were sustained improvements in HRQL scores and whether there were specific determinants of HQRL score over the course of therapy. Therefore, we studied a new and distinct population of participants with a larger sample size (n = 57) and a longer follow-up of 24-months and examined characteristics associated with worse or improved HQRL scores.

## Methods

### Participants

Questionnaires were distributed randomly to the caregivers of 57 children (demographic and clinical characteristics of patient population are described in Table 1) enrolled in food allergen IT between April 1 2012 and October 1 2013. IT was performed similarly in all 57 children, as per a published protocol [9], under Stanford IRB approval. Briefly, participants older than 4 years were eligible for inclusion if they had proven sensitivity to one or more food allergen documented by both a positive skin prick test specific IgE as well as positive allergic reaction in a double-blind placebo-controlled oral food challenge (DBPCFC) up to a cumulative dose of 182 mg. All allergic reactions during the course of IT were described as per Bock’s criteria for each food allergen [14].

**Table 1:** **Characteristics of patient population**

	Baseline	6-month follow-up time point	12-month follow-up time point	18-month follow-up time point	24-month follow-up time point
**Number of subjects**	57	57	45	41	37
**Median Age in years (range)**	9 (4 – 18)	9 (4 – 18)	9 (4 – 18)	9 (4 - 18)	9 (4 – 18)
**Male**	35 (61%)	35 (61%)	25 (56%)	24 (58%)	22 (59%)
**Ethnicity**					
Caucasian	30 (53%)	30 (53%)	25 (55%)	23 (56%)	20 (54%)
Asian	18 (32%)	17 (32%)	13 (29%)	11 (27%)	11 (30%)
Indian	5 (9%)	5 (9%)	5 (11%)	5 (12%)	5 (13%)
Hispanic	3 (5%)	3 (5%)	2 (4%)	2 (5%)	1 (3%)
African-American	1 (2%)	1 (2%)	0 (0%)	0 (0%)	0 (0%)
**Coexisting atopic disease**					
Asthma	37 (65%)	37 (65%)	27 (60%)	37 (59%)	21 (57%)
Allergic Rhinitis	30 (52%)	30 (52%)	25 (55%)	20 (50%)	20 (54%)
Atopic Dermatitis	17 (30%)	17 (30%)	13 (30%)	12 (30%)	12 (32%)
**Number of allergens**					
1	24 (42%)	24 (42%)	14 (31%)	13 (32%)	14 (38%)
2	13 (23%)	13 (23%)	13 (29%)	11 (27%)	8 (22%)
3	8 (14%)	8 (14%)	8 (18%)	8 (19%)	8 (22%)
4	4 (7%)	4 (7%)	3 (6%)	3 (7%)	2 (5%)
5	8 (14%)	8 (14%)	6 (13%)	5 (12%)	5 (13%)
**Allergens**					
Peanut	48 (84%)	47 (84%)	38 (84%)	33 (80%)	31 (84%)
Cashew	17 (30%)	17 (30%)	15 (33%)	13 (32%)	17 (46%)
Walnut	12 (21%)	12 (21%)	11 (24%)	10 (24%)	9 (24%)
Egg	12 (21%)	12 (21%)	10 (22%)	11 (27%)	8 (22%)
Pecan	10 (17%)	10 (17%)	9 (20%)	8 (19%)	8 (22%)
Milk	8 (14%)	8 (14%)	7 (15%)	7 (17%)	6 (16%)
Almond	6 (10%)	6 (10%)	5 (11%)	5 (12%)	5 (13%)
Sesame	4 (7%)	4 (7%)	4 (9%)	4 (10%)	4 (11%)
Hazelnut	3 (5%)	3 (5%)	3 (7%)	2 (5%)	1 (3%)
Shellfish	3 (5%)	3 (5%)	1 (2%)	1 (2%)	1 (3%)

Demographics and clinical characteristics of patients are shown at baseline, 6-month follow-up, 12-month follow-up, 18-month follow-up, and 24-month follow-up time point.

### Study intervention

The intervention used have been previously described in detail [9]. Briefly, subjects meeting inclusion criteria were started on a daily dose of up to 5 multiple food allergens combined in an equivalent ratio (1:1:1:1:1) based on food protein content. Participants underwent an initial dose escalation starting at 0.1 mg of total food protein up to a maximum of 6 mg if tolerated. The maximal tolerated dose determined daily home dose that was increased by 25% increments every other week at our research clinic based on dose tolerability. Participants took a median of 85 weeks to reach their maintenance dose of 4000 mg protein per food. At 24 months, participants were on maintenance doses of their food allergen IT (4 g of each allergen daily). No patient was treated with omalizumab.

### Questionnaire

A validated Food Allergy Quality of Life – Parental Burden (FAQL-PB) questionnaire was used to assess HRQL [15]. Originally validated in the United States by Cohen et al., it is a FA-specific HRQL questionnaire that measures parental burden related to having a child with FA. It consists of 17 questions that are answered on a 7-point scale and related to two main specific aspects such as social and dietary limitation and food-related emotional impact (Additional file 1: Table S1). The same parent (12 fathers and 45 mothers) was asked to answer the questionnaire at baseline and at 6-month, 12-month, 18-month, and 24-month time points on IT.

### Statistics

Statistical analysis was performed using t test with Welch’s correction and the Mann-Whitney test for unpaired variables and Wilcoxon for paired variables, as appropriate. All tests were two-tailed and significance was set at p-value less than 0.05.

The MID (minimal clinically important difference) was used to assess the clinical relevance. Defined as the smallest change in HRQL score that participants perceive as clinically important, it has been estimated to be approximately 0.5 for scores graded on a 7-point scale in several HRQL questionnaires, including questionnaires determining parental quality of life [16–20].

All statistical analyses and graphing were carried out using GraphPad Prism software (GraphPad Software, San Diego, CA).

## Results

All randomly chosen 57 caregivers filled out the questionnaire for baseline and at least one follow-up time-point. Particularly, 57 answered for 6-month follow-up time-point and baseline, 45 for 12-month follow-up time-point and baseline, 41 for 18-month follow-up time-point and baseline, and 37 for 24-month follow-up time-point and baseline (Table 1). 37 caregivers completed all 5 time-points.

**Figure 1:**
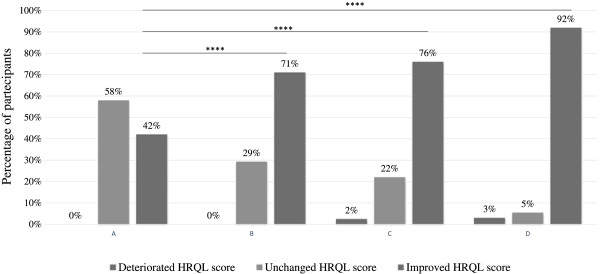
**Percentages of participants with deterioration, no change, or improvement in the HRQL score.** Percentages of participants whose HRQL score deteriorated (change >0.5), remained unchanged (change between -0.5 and 0.5), or improved (change < -0.5) at **(A)** 6-month follow-up, **(B)** 12-month follow-up, **(C)** 18-month follow-up, and **(D)** 24-month follow-up from baseline. ****p <0.0001.

The HRQL scores improved significantly (change < - 0.5, p <0.0001) between each time point and baseline. The percentages of caregivers documenting improvements in HRQL scores of their children between 24-month and baseline were progressively higher over time (42%, 71%, 76% and 92% respectively at the 6-month, 12-month, 18-month and 24-month follow-up time point compared to baseline). The percentage of those whose HRQL score deteriorated (change > +0.5) were 0%, 0%, 2%, and 3% respectively at the 6-month, 12-month, 18-month and 24-month follow-up time point compared to baseline (Figure 1). The percentages of those whose HRQL score remained unchanged (change between -0.5 and 0.5) were 58%, 29%, 22% and 5%, respectively at the 6-month, 12-month, 18-month and 24-month follow-up time point compared to baseline (Figure 1).

**Figure 2:**
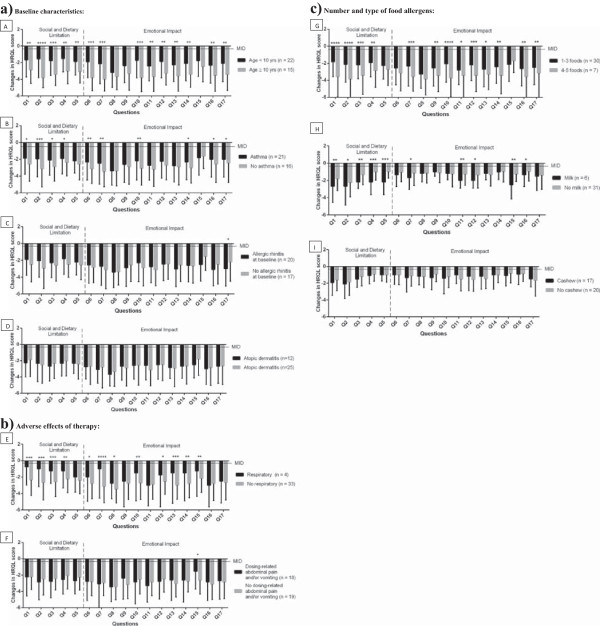
**Changes in individual FAQL-PB question scores at 24-month follow-up time point from baseline for: a) baseline characteristics: (A) age, (B) asthma at baseline, (C) allergic rhinitis at baselineand (D) atopic dermatitis at baseline; b) adverse effects of therapy: (E) respiratory-related allergic reactions and (F) dosing- related abdominal pain and/or vomiting; c) number and type of food allergens: (G) 1-3 versus 4-5 foods; (H) milk; (I) cashew.** Changes in individual FAQL-PB question scores are shown between caregivers of: **(A)** patients <10 years old and patients ≥10 years old; **(B)** patients with asthma and patients without asthma at baseline; **(C)** patients with allergic rhinitis and patients without allergic rhinitis at baseline; **(D)** patients with atopic dermatitis and without atopic dermatitis at baseline; **(E)** patients with at least one documented by a physician dosing- related respiratory adverse reaction (wheezing and/or cough) and patients without those; **(F)** patients with abdominal pain and/or vomiting and patients without abdominal pain and/or vomiting; **(G)** patients in treatment with 1-3 food allergens and patients in treatment with 4-5 food allergens; **(H)** patients in treatment with milk and patients not in treatment with milk; **(I)** patients in treatment with cashew and patients not in treatment with cashew. *p <0.05, **p <0.01, ***p <0.001, ****p <0.0001. Bars without asterisks represent non-significant changes.

We then examined the effect of baseline characteristics on the degree of HRQL improvement on IT at 24 months. Baseline demographic characteristics of the study participants are shown in Table 1. To determine if changes in the HRQL score were associated with age, we divided 35 subjects into two groups: age <10 years (n =22) and age ≥10 years (n =15). Caregivers of patients in the ≥10 years age group had significantly more improvement compared to those in the <10 years age group (p <0.0001) (Figure 2A). Within the FAQL-PB questionnaire of 17 questions, the questions related to choice of restaurant (p <0.0001), planning to participate in social activities with others involving food on one side (p <0.001), and about anxiety relating to food allergy (p <0.001) were the most significantly different for ages greater than or equal to 10 years (Figure 2A). There was no association between the gender of the participants and the change in HRQL score (Additional file 2: Figure S1A).

Next, we investigated the role of co-morbid allergic disorders at baseline. HRQL scores of participants without asthma (n =21) had significantly more improvement (p <0.01) when compared to participants with baseline asthma (n =16). This difference was detected most significantly in the questions related to the choice of restaurant (p <0.001), the risk of not overcoming food allergy (p <0.01), and the burden for the child because of their own food allergy (p <0.01) (Figure 2B). Baseline co-morbid allergic rhinitis (Figure 2C) and atopic dermatitis (Figure 2D) did not significantly affect changes in HRQL score.

We hypothesized that certain allergic events during IT, like wheezing, would affect the outcome of HRQL score. We found that dose-related respiratory allergic reactions (wheezing and cough) were associated (p <0.0001) with less improvement in the HRQL score. Questions related to the trouble of overcoming food allergy (p <0.0001) and worry of not being able to help their own child in case of allergic reaction to food (p <0.001) and dietary-social limitations (planning holiday/vacation, choicing a restaurant, participating in social activities with others involving food, p <0.001) were the most significantly different for dosing-related respiratory allergic reactions (Figure 2E). All subjects with respiratory allergic reactions during dosing were asthmatic at baseline. No association was detected with dosing-related abdominal pain and vomiting (Figure 2F), allergic rhinitis (Additional file 2: Figure S1B), or skin reactions (Additional file 2: Figure S1C).

We also analyzed if changes in HRQL score varied based on number or type of food allergens used during desensitization. We found that improvement in HRQL scores of subjects treated with 4-5 food allergens (n =6) were significantly greater compared to those treated with 1-3 foods (p <0.0001). This difference were detected most significantly in the questions related to sadness regarding the child’s burden related to food allergy (p <0.0001), planning holiday/vacation (p <0.0001) and choice of a restaurant (p <0.0001), as shown in Figure 2G. There were no significant differences in the changes of HRQL score between subjects treated with a single food allergen (n =14) versus multiple food allergens (n =23), (Additional file 2: Figure S1D), and between subjects treated with one or two food allergens (n =15) versus more than two food allergens (n =22), (Additional file 2: Figure S1E).

Caregivers’ HRQL scores of 6 subjects treated with cow’s milk had a significantly greater improvement (p =0.006) than subjects treated with other food allergens (n =31). This improvement was detected most significantly in the questions regarding the concern about child’s nutrition (p <0.001), and the need to spend extra time preparing meals (i.e., label reading, extra time shopping, preparing meals, etc.) (p <0.001) and to take special precautions before going out of the home with their child (p <0.001), (Figure 2H). Significant differences were not found in HRQL score change between the presence (n =17) or absence (n =20) of cashew in the food allergens used in treatment (Figure 2I).

## Discussion

Using a quality of life questionnaire specifically validated for food allergy, we examined the effects of IT on the quality of life of caregivers of patients suffering from single and multiple food allergies. In addition to following these participants for 24 months (longer than any previously published study to our knowledge), we have determined characteristics that could possibly assist in identifying those who may psychologically benefit the most from IT.

The improvement we saw in HRQL scores on IT is consistent with previously published data [13, 18, 19]. Recently, the relevance of IT as a long-term intervention for food allergy has come into question, as studies have shown limited rates of sustained unresponsiveness with IT [21]. This study, consistent with our previously published study [13], shows that IT provides significant improvement in caregiver HRQL in participants who continue to take IT. Long-term follow-up is critical to test net improvements over time. We plan to continue to follow these caregivers and participants to allow future studies on HRQL.

The significantly greater improvement in caregiver HRQL in patients older than 10 years raises the interesting question of how to possibly prioritize some age groups for IT. This question needs to be studied long term by utilizing validated food allergy quality-of-life questionnaires for different age groups (FAQL-teen, FAQLQ-adult, etc) to assess the effect of IT on participants themselves.

We also found less improvement in caregiver HRQL in subjects with baseline asthma. Clearly food-induced respiratory symptoms should be managed differently from asthma exacerbations triggered by other common triggers, with injectable epinephrine as the treatment of choice as opposed to inhaled beta-agonists. Recent studies demonstrate not only that food allergy and asthma often coexist but also that having these co-morbid conditions worses prognosis: children with food allergies and asthma are more likely to have near-fatal or fatal allergic reactions to food (among which respiratory symptoms) and more likely to have severe asthma [22] with negative impact on quality of life. This may explain why these patients in particular were precluded from greater improvements in HRQL [23], as we found that caregivers of patients with dose-related respiratory allergic reactions had less improvement in HRQL. All patients with dose-related respiratory allergic reactions were asthmatic at baseline. As respiratory allergic events are more tied to life-threatening events, they may be particularly burdensome [24], increasing parents’ sense of anxiety about future reactions.

Compared to patients with single food allergies, patients with multiple food allergies experience a greater decrease in quality of life [15, 23], are more likely to suffer from dietary deficiencies [25] and are less prone to spontaneously outgrow their food allergies [26]. Building on our previous study [13], which found that mIT improves caregiver HRQL in children with multiple food allergies, we analyzed a larger study population in this study and found that HRQL score improves more in subjects treated with 4-5 food allergens compared to those treated with <4 food allergens (p <0.0001) (Figure 2G). As a similar difference was not seen between single versus multiple food allergen therapy (Additional file 2: Figure S1D), and <2 versus ≥2 food allergen therapy (Additional file 2: Figure S1E), it is possible that patients with multiple food allergies must be treated with at least a certain number of food allergens to achieve a greater improvement in caregiver HRQL.

There are limitations to our study. Although our sample size was larger than our previous study, it still lacked power to detect significant differences between some types of food allergens and between quality of allergic reactions (severe, moderate vs. mild). There was no untreated control group. However, our hypothesis was best tested by comparisons against baseline to allow for each caregiver’s scoring and perception to be similarly tested.

In conclusion, the results reported here support our previous findings [13] that IT could possibly lead to significant and long-term improvements in caregiver HRQL. Furthermore, we were able to identify baseline characteristics that could potentially help identify patients who would benefit the most from IT. Validated measures of quality of life should be included in future food allergy clinical trials.

## Consent

Written informed consent was obtained from the patient’s guardian/parent/next of kin for the publication of this report and any accompanying images.

## Electronic supplementary material

Additional file 1: Table S1: Food Allergy Quality of Life – Parental Burden Questionnaire. (DOC 55 KB)

Additional file 2: Figure S1: Changes in individual FAQL-PB question scores at 24-month follow-up time point from baseline for: (A) gender; (B) dosing- related allergic rhinitis; (C) ( dosing- related skin reactions; number of food allergens: (D) single food versus multiple foods and (E) 1-2 versus >2 foods. Changes in individual FAQL-PB question scores are shown between caregivers of: (A) male versus female patients; (B) patients with versus patients without dosing related allergic rhinitis; (C) - patients with versus patients without dosing related skin reactions; (D) patients in treatment with single food allergen versus caregivers of patients in treatment with multiple food allergens; (E) patients in treatment with 1-2 food allergens versus caregivers of patients in treatment with more than 2 food allergens. All the comparisons between groups were n. s. (not significant). *p <0.05. Bars without asterisks represent non-significant changes. (PDF 457 KB)

## Abbreviations

FA: Food allergy
IT: Immunotherapy
mIT: Immunotherapy with multiple food allergens simultaneously
HRQL: Health-related quality of life
FAQL-PB: Food allergy quality of life – parental burden
MID: Minimal clinically important difference.
