# Supplementary material for: Two year effects of food allergen immunotherapy on quality of life in caregivers of children with food allergies
Source: Allergy Asthma Clin Immunol. 2014 Nov 25;10(1):57. doi: 10.1186/1710-1492-10-57 (PMC4363059; doi:10.1186/1710-1492-10-57)
Supplement: Supplementary file 1 — Additional file 1: Table S1: Food Allergy Quality of Life – Parental Burden Questionnaire. (DOC 55 KB) [file 13223_2014_525_MOESM1_ESM.doc]

**Additional file 1: Table S1**

**Food Allergy Quality of Life – Parental Burden Questionnaire.**

1. If you and your family were planning a holiday/vacation, how much would your choice of vacation be limited by your child’s food allergy?

| 0 –  Not limited | 1 –  Hardly limited at all | 2 –  Somewhat limited | 3 – Moderately limited | 4 –  Quite a bit limited | 5 –  Very limited | 6 –  Extremely limited |
| --- | --- | --- | --- | --- | --- | --- |

1. If you and your family were planning to go to a restaurant, how much would your choice of a restaurant be limited by your child’s food allergy?

| 0 –  Not limited | 1 –  Hardly limited at all | 2 –  Somewhat limited | 3 – Moderately limited | 4 –  Quite a bit limited | 5 –  Very limited | 6 –  Extremely limited |
| --- | --- | --- | --- | --- | --- | --- |

1. If you and your family were planning to participate in social activities with others involving food (e.g., parties, holiday, etc.), how limited would your ability to participate in social activities that involve food be because of your child’s food allergy?

| 0 –  Not limited | 1 –  Hardly limited at all | 2 –  Somewhat limited | 3 – Moderately limited | 4 –  Quite a bit limited | 5 –  Very limited | 6 –  Extremely limited |
| --- | --- | --- | --- | --- | --- | --- |

1. In the past week, how troubled have you been by your need to spend extra time preparing meals (i.e., label reading, extra time shopping, preparing meals, etc.) due to your child’s food allergy?

| 0 –  Not troubled | 1 –  Hardly troubled at all | 2 –  Somewhat troubled | 3 –  Moderately troubled | 4 –  Quite a bit troubled | 5 –  Very troubled | 6 –  Extremely troubled |
| --- | --- | --- | --- | --- | --- | --- |

1. In the past week, how troubled have you been about your need to take special precautions before going out of the home with your child because of their food allergy?

| 0 –  Not troubled | 1 –  Hardly troubled at all | 2 –  Somewhat troubled | 3 –  Moderately troubled | 4 –  Quite a bit troubled | 5 –  Very troubled | 6 –  Extremely troubled |
| --- | --- | --- | --- | --- | --- | --- |

1. In the past week, how troubled have you been by anxiety relating to your child’s food allergy?

| 0 –  Not troubled | 1 –  Hardly troubled at all | 2 –  Somewhat troubled | 3 – Moderately troubled | 4 –  Quite a bit troubled | 5 –  Very troubled | 6 –  Extremely troubled |
| --- | --- | --- | --- | --- | --- | --- |

1. In the past week, how troubled have you been that your child may not overcome their food allergy?

| 0 –  Not troubled | 1 –  Hardly troubled at all | 2 –  Somewhat troubled | 3 – Moderately troubled | 4 –  Quite a bit troubled | 5 –  Very troubled | 6 –  Extremely troubled |
| --- | --- | --- | --- | --- | --- | --- |

1. In the past week, how troubled have you been by the possibility of, or actually leaving your child in the care of others because of their food allergy?

| 0 –  Not troubled | 1 –  Hardly troubled at all | 2 –  Somewhat troubled | 3 – Moderately troubled | 4 –  Quite a bit troubled | 5 –  Very troubled | 6 –  Extremely troubled |
| --- | --- | --- | --- | --- | --- | --- |

1. In the past week, how troubled have you been by frustration over others’ lack of appreciation for the seriousness of food allergy?

| 0 –  Not troubled | 1 –  Hardly troubled at all | 2 –  Somewhat troubled | 3 – Moderately troubled | 4 –  Quite a bit troubled | 5 –  Very troubled | 6 –  Extremely troubled |
| --- | --- | --- | --- | --- | --- | --- |

1. In the past week, how troubled have you been by sadness regarding the burden your child carries because of their food allergy?

| 0 –  Not troubled | 1 –  Hardly troubled at all | 2 –  Somewhat troubled | 3 – Moderately troubled | 4 –  Quite a bit troubled | 5 –  Very troubled | 6 –  Extremely troubled |
| --- | --- | --- | --- | --- | --- | --- |

1. In the past week, how troubled have you been about your child’s attending school, camp, daycare or other group activity with children because of their food allergy?

| 0 –  Not troubled | 1 –  Hardly troubled at all | 2 –  Somewhat troubled | 3 – Moderately troubled | 4 –  Quite a bit troubled | 5 –  Very troubled | 6 –  Extremely troubled |
| --- | --- | --- | --- | --- | --- | --- |

1. In the past week, how troubled have you been by your concerns for your child’s health because of their food allergy?

| 0 –  Not troubled | 1 –  Hardly troubled at all | 2 –  Somewhat troubled | 3 – Moderately troubled | 4 –  Quite a bit troubled | 5 –  Very troubled | 6 –  Extremely troubled |
| --- | --- | --- | --- | --- | --- | --- |

1. In the past week, how troubled have you been with the worry that you will not be able to help your child if they have an allergic reaction to food?

| 0 –  Not troubled | 1 –  Hardly troubled at all | 2 –  Somewhat troubled | 3 – Moderately troubled | 4 –  Quite a bit troubled | 5 –  Very troubled | 6 –  Extremely troubled |
| --- | --- | --- | --- | --- | --- | --- |

1. In the past week, how troubled have you been with the worry that your child will not have a normal upbringing because of their food allergy?

| 0 –  Not troubled | 1 –  Hardly troubled at all | 2 –  Somewhat troubled | 3 – Moderately troubled | 4 –  Quite a bit troubled | 5 –  Very troubled | 6 –  Extremely troubled |
| --- | --- | --- | --- | --- | --- | --- |

1. In the past week, how troubled have you been about concerns for your child’s nutrition because of their food allergy?

| 0 –  Not troubled | 1 –  Hardly troubled at all | 2 –  Somewhat troubled | 3 – Moderately troubled | 4 –  Quite a bit troubled | 5 –  Very troubled | 6 –  Extremely troubled |
| --- | --- | --- | --- | --- | --- | --- |

1. In the past week, how troubled have you been with issues concerning your child being near others while eating because of their food allergy?

| 0 –  Not troubled | 1 –  Hardly troubled at all | 2 –  Somewhat troubled | 3 – Moderately troubled | 4 –  Quite a bit troubled | 5 –  Very troubled | 6 –  Extremely troubled |
| --- | --- | --- | --- | --- | --- | --- |

1. In the past week, how troubled have you been with being frightened by the thought that your child will have a food allergic reaction?

| 0 –  Not troubled | 1 –  Hardly troubled at all | 2 –  Somewhat troubled | 3 – Moderately troubled | 4 –  Quite a bit troubled | 5 –  Very troubled | 6 –  Extremely troubled |
| --- | --- | --- | --- | --- | --- | --- |
