# Supplementary figures and images for: Two year effects of food allergen immunotherapy on quality of life in caregivers of children with food allergies
Source: Allergy Asthma Clin Immunol. 2014 Nov 25;10(1):57. doi: 10.1186/1710-1492-10-57 (PMC4363059; doi:10.1186/1710-1492-10-57)

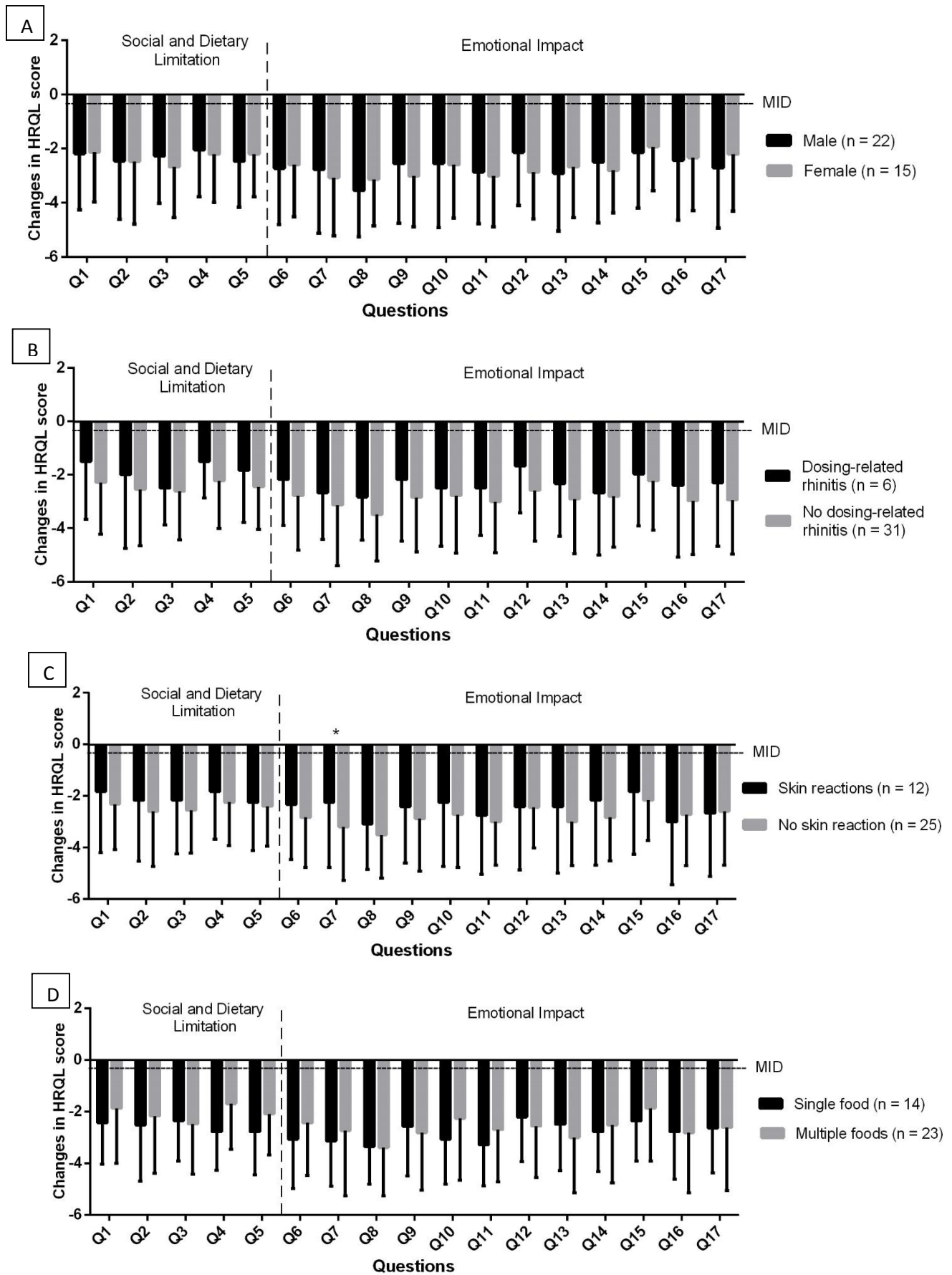

F

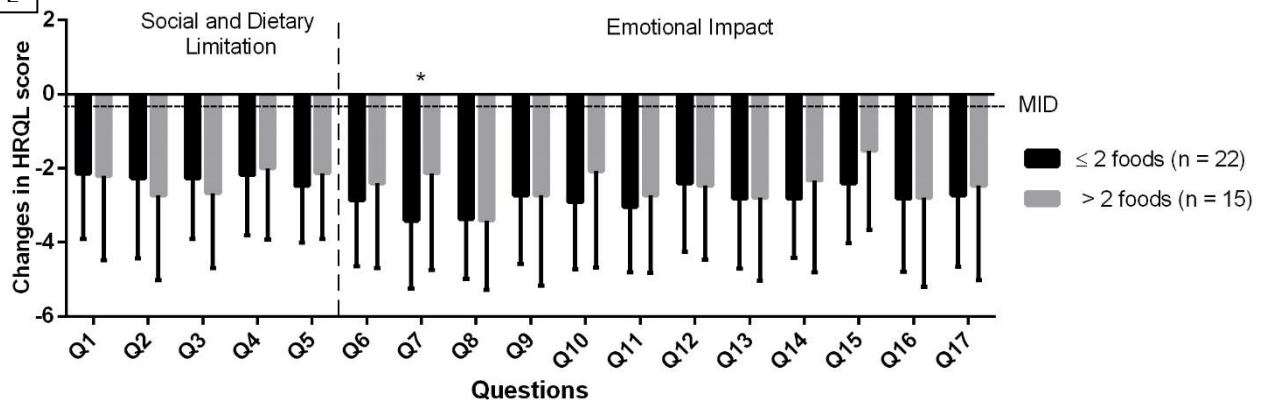

Supplement: Supplementary file 2 — Additional file 2: Figure S1: Changes in individual FAQL-PB question scores at 24-month follow-up time point from baseline for: (A) gender; (B) dosing- related allergic rhinitis; (C) ( dosing- related skin reactions; number of food allergens: (D) single food versus multiple foods and (E) 1-2 versus >2 foods. Changes in individual FAQL-PB question scores are shown between caregivers of: (A) male versus female patients; (B) patients with versus patients without dosing related allergic rhinitis; (C) - patients with versus patients without dosing related skin reactions; (D) patients in treatment with single food allergen versus caregivers of patients in treatment with multiple food allergens; (E) patients in treatment with 1-2 food allergens versus caregivers of patients in treatment with more than 2 food allergens. All the comparisons between groups were n. s. (not significant). *p <0.05. Bars without asterisks represent non-significant changes. (PDF 457 KB) [file 13223_2014_525_MOESM2_ESM.pdf]
